# Isothermal folding of a light-up bio-orthogonal RNA origami nanoribbon

**DOI:** 10.1038/s41598-018-25270-6

**Published:** 2018-05-03

**Authors:** Emanuela Torelli, Jerzy Wieslaw Kozyra, Jing-Ying Gu, Ulrich Stimming, Luca Piantanida, Kislon Voïtchovsky, Natalio Krasnogor

**Affiliations:** 1 Interdisciplinary Computing and Complex BioSystems (ICOS), School of Computing Science, Centre for Synthetic Biology and Bioeconomy (CSBB), Centre for Bacterial Cell Biology (CBCB), Newcastle University, Newcastle upon Tyne, NE4 5TG United Kingdom; 2 School of Chemistry, Newcastle University, Newcastle upon Tyne, NE1 7RU United Kingdom; 3 Department of Physics, Durham University, Durham, DH1 3LE United Kingdom

## Abstract

RNA presents intringuing roles in many cellular processes and its versatility underpins many different applications in synthetic biology. Nonetheless, RNA origami as a method for nanofabrication is not yet fully explored and the majority of RNA nanostructures are based on natural pre-folded RNA. Here we describe a biologically inert and uniquely addressable RNA origami scaffold that self-assembles into a nanoribbon by seven staple strands. An algorithm is applied to generate a synthetic De Bruijn scaffold sequence that is characterized by the lack of biologically active sites and repetitions larger than a predetermined design parameter. This RNA scaffold and the complementary staples fold in a physiologically compatible isothermal condition. In order to monitor the folding, we designed a new split Broccoli aptamer system. The aptamer is divided into two nonfunctional sequences each of which is integrated into the 5′ or 3′ end of two staple strands complementary to the RNA scaffold. Using fluorescence measurements and in-gel imaging, we demonstrate that once RNA origami assembly occurs, the split aptamer sequences are brought into close proximity forming the aptamer and turning on the fluorescence. This light-up ‘bio-orthogonal’ RNA origami provides a prototype that can have potential for *in vivo* origami applications.

## Introduction

A plethora of self-assembled DNA origami and hybrid RNA-DNA origami nanostructures have been synthesized using the basic principle of Watson-Crick base pairing^1–9^. The scaffolded DNA origami technique involves short oligonucleotide staple strands which direct the folding of a long scaffold strand sequence into a pre-programmed nanostructure^6^ with well-defined shape, dimension and functional properties^10^.

As an interesting alternative, nanostructures can be self-assembled from RNA instead of DNA. The advantages of RNA structures include their potential applications *in vivo*, their expression in large quantities, the formation of stable interactions^11^ and the control of gene expression^12^. Despite RNA’s functional capacities, the synthesis of RNA nanostructures via scaffold and staple strands is underdeveloped and still lacking. While significant advances have been made in DNA origami synthesis, the design and realization of RNA origami has been reported only recently^13^. Indeed, most of the RNA nanostructures have been developed under different approaches based on the self-assembly of building blocks such as loops and motifs^14–16^. In this context, several RNA sequences such as packaging RNA (pRNA) from bacteriophage ϕ29^17–20^, transfer RNA (tRNA)^21^ and an engineered modular ribozyme^22^ were used to guide the self-assembly of RNA nanoparticles^14,23^.

In contrast to the above mentioned strategies used for the construction of RNA nanoparticles, Yingling and Shapiro^24^ introduced a shape-based computational approach to design RNA hexagonal nanoring from two building blocks using stable RNAIi/RNAIIi loop-loop noncovalent intermolecular interactions. In this regard, a computational-based approach promoted the *in silico* design of a three dimensional cubic RNA-based scaffold that was self-assembled in a one-pot process. Instead of prefolded RNA, the nanostructure required relatively short RNA sequences and allowed selective point modifications^25–27^.

Recently, RNA nanostructures were also obtained with different tools and techniques imported from DNA nanotechnology, such as the use of the double cross over (DX) motifs and the DNA origami design. For instance, a tile-based assembly approach was investigated to assemble micron-size RNA scaffolds and a DX DNA tile motif was adapted to design DX RNA tiles that formed lattices and tubular structures via programmed sticky end interactions^28,29^. Taking into account the powerful origami technique, Geary *et al*.^30^ showed the cotrascriptional folding of an artificially designed single stranded RNA (ssRNA) into a RNA tile: at the end of the RNA tile synthesis, the tiles were released and assembled into hexagonal lattices through kissing loop interactions. In contrast to the traditional origami technique, this strategy used RNA modules such as kissing loops to replace the staple strand role^30^. In the same year, a 7-helix bundled RNA tile and a 6-helix bundled RNA origami tube were designed and prepared from single stranded RNA scaffold and multiple staple RNA strands by employing for the first time a direct extension of the DNA origami strategy^13^.

Here we take into account the large gap between DNA origami and RNA origami development and inspired by a bottom-up origami toolkit, we present a light-up biologically inert (i.e. ‘bio-orthogonal’) RNA origami able to fold at constant temperature after an initial denaturation step. In our recent work^8^, a square DNA origami and a triangle RNA-DNA hybrid origami were synthesized using ‘bio-orthogonal’ and uniquely addressable De Bruijn scaffold sequences (DBS). Unlike biologically derived scaffolds like M13mp18 or pUC19, these DNA and RNA ‘bio-orthogonal’ scaffolds do not contain genetic information, restriction enzyme sites or ambiguity in the addressability, making them candidates for future *in vivo* applications^8^. In this work RNA staple strands promoted the folding of a short ‘bio-orthogonal’ RNA scaffold sequence into a nanoribbon shaped structure: after an initial denaturation step, the self-assembly occured at physiological temperature (37 °C) within minutes. RNA origami assembly was verified by gel assay, atomic force microscopy (AFM) and using a new split Broccoli aptamer system able to bind the specific fluorophore only after the folding process. The Broccoli aptamer^31^ was divided into two non-functional RNA sequences each of which were integrated into two distinct RNA staple strands complementary to the scaffold sequence. Once the RNA origami was assembled, the split aptamer sequences were reassociated to form the functional binding site and the fluorescence was clearly restored.

Herein, we investigate and combine three different aspects: i) ‘bio-orthogonality’, ii) physiologically compatible folding at 37 °C and iii) assembly monitoring via a new split Broccoli RNA aptamer system. These characteristics are important from the perspective of RNA origami expression and folding in living cells. Our RNA origami nanoribbon opens the way to new potential platform for future *in vivo* origami applications when genetically encoded and transcribed RNA are used. RNA can control many cellular functions and the creation of new tools that allow the programmable control of gene expression is a fundamental goal in synthetic biology. In this perspective, RNA origami can be used as organelle-like structure able to act as regulatory machine.

## Results and Discussion

### Synthetic RNA scaffold design

**Figure 1:**
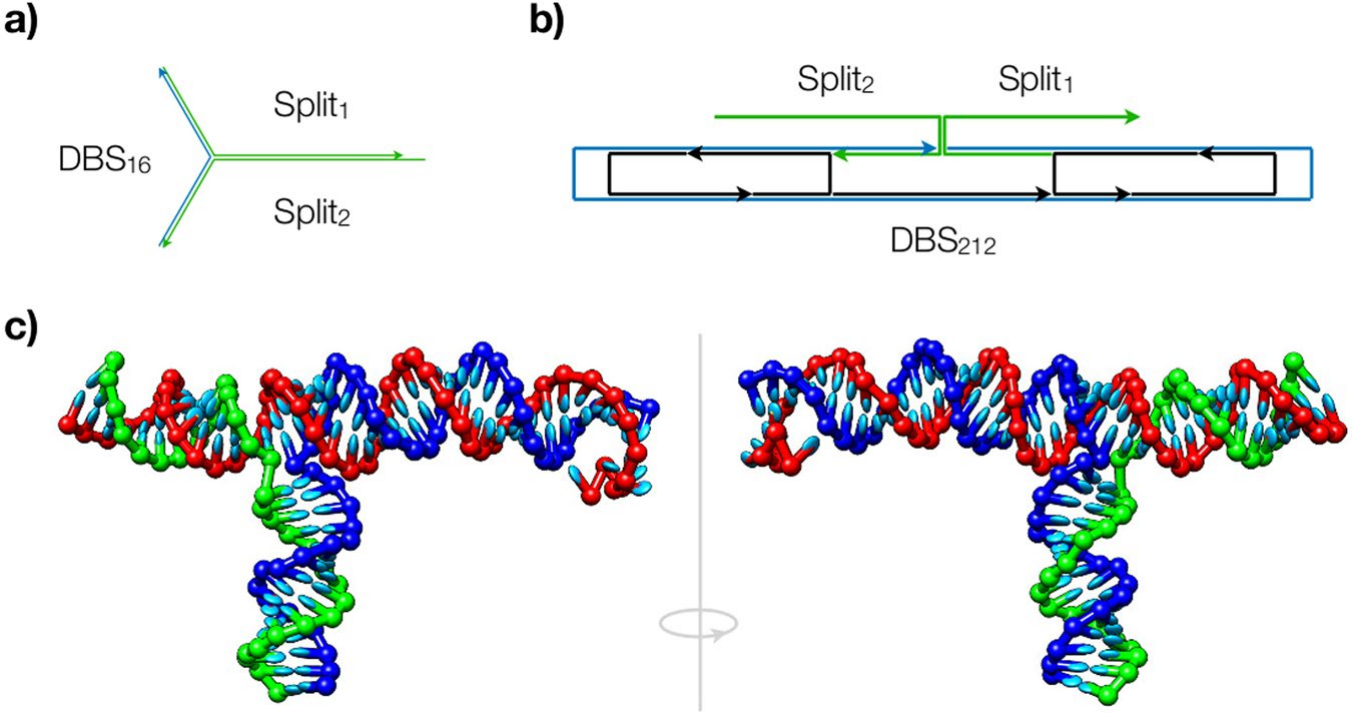
The overview of the designs used in this study. (**a**) Split1, Split2 and the complementary DBS forming a three-way junction; (**b**) RNA origami ribbon decorated with the Split1 and Split2 staples which bind to the opposite ends of the DBS scaffold; (**c**) visualisation of the three-way junction MFE structure as simulated by oxRNA coarse-grained model (DBS in red, Split1 in blue, Split2 in green).

Previously we descibed the creation of ‘bio-orthogonal’ scaffolds for RNA origami systems^8^. Here, we use a similar method to obtain candidate sequences that interface with the Broccoli RNA aptamer system. The first synthetic scaffold was constructed to act solely as a platform for the split aptamer. In this design, the 32 nt-long De Bruijn sequence forms a three-way junction with the complementary domains integrated with the split aptamer, allowing for its reconstruction (Fig. 1a). This enables measurement of the baseline fluorescent signal in absence and presence of the scaffold.

Also, the formation of the three-way junction was simulated using a coarse-grained model of RNA. In our simulation, the system is initialised with the synthetic scaffold hybridized to the two aptamer domains and run until equilibrium is reached. In this state, one can observe the reconstructed aptamer (Fig. 1c) which agrees partially with the mFold prediction (the main discrepancy being absence of bulge and internal loops).

Furthermore, to test the aptamer system integrated into the RNA origami, we constructed a 212 nt long DBS scaffold which was designed to fold into a ribbon; 27 nm × 5 nm in size. In this design, 5 staple strands are folding the scaffold and 2 staples (i.e. split aptamers) are used to ‘seal’ the folding (Fig. 1b). Here, the main objective was to characterise the Broccoli RNA aptamer as a potential monitoring component for the RNA origami folding. Namely, correct folding of the origami should promote the reconstruction of the aptamer, and thus, result in an increase of the fluorescence signal.

### Split Broccoli aptamer system and fluorescence activation

The Spinach aptamer sequence, an RNA mimic of green fluorescent protein, opened the way to a novel class of RNA sequences able to emit fluorescence in the presence of a specifically provided fluorophore molecule^32^. The Spinach technology has been used to monitor different small molecules in live cells^33–38^, transcription *in vitro* and *in vivo*^39,40^ and RNA assembly^41^, to image mRNA^42^, to design genetic circuits^43^ and to measure ribozyme self-cleavage activity^44^.

Considering the usefulness of Spinach, more recently a new smallest light-up aptamer, called Broccoli, has been described^31^. A unique characteristic of this aptamer is its short size that can improve its versatility when used as a tag. Moreover Broccoli aptamer shows robust folding and green fluorescence in physiological conditions.

**Figure 2:**
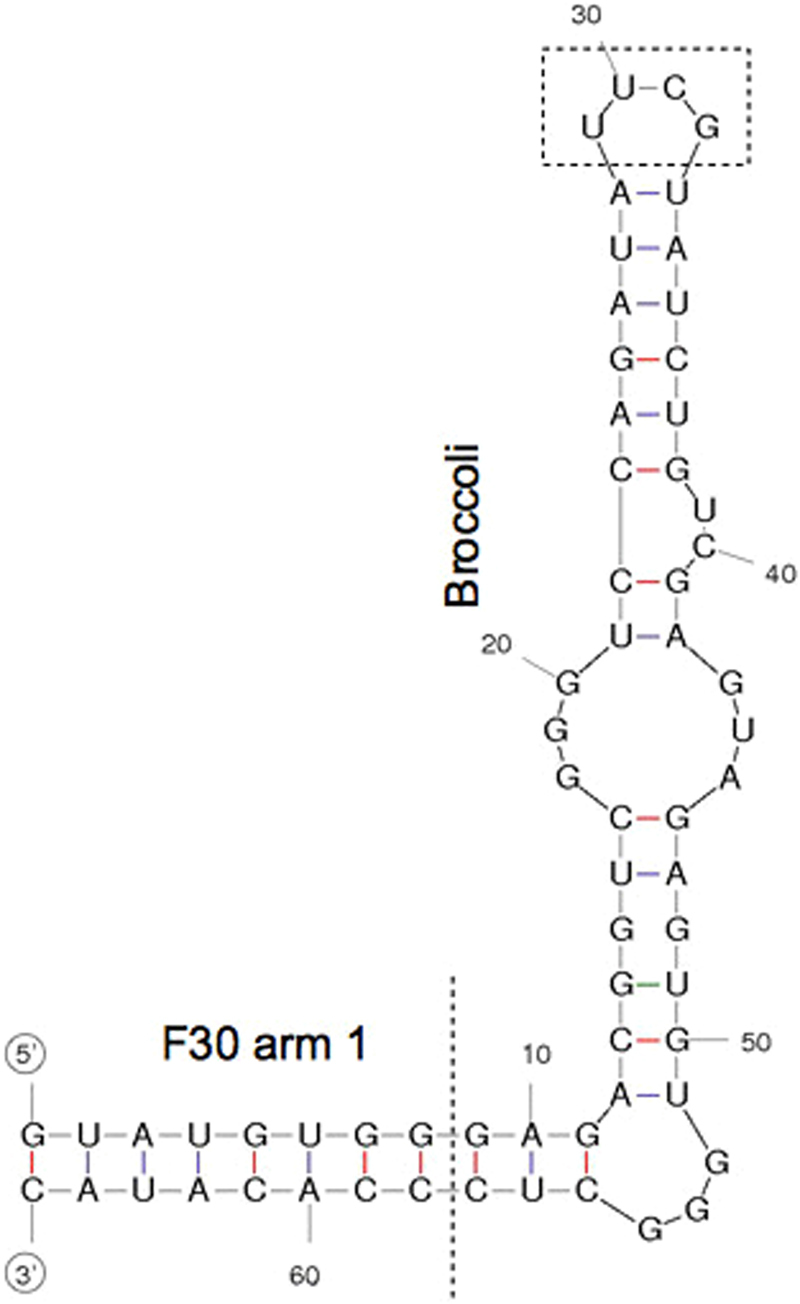
Sequence and mFold predicted structure of Broccoli aptamer with part of the F30 arm 1 used in this study. To obtain the split system, two sequences complementary to the DBS scaffold were added at the 5′ and 3′ ends (circles), and the 4-nt terminal stem loop (rectangle) was removed.

Taking all these features together and considering the introduction of the brighter, highly selective and non-cytotoxic DFHBI-1T fluorophore, we designed and tested a new RNA split Broccoli aptamer system (see Supplementary Fig. S1): in the presence of a complementary Split1/Split2 DBS, the two RNA sequences (called Split1 and Split2) hybridized and the fluorescence was activated. In a previous report on Broccoli aptamer, Filonov *et al*.^45^ reengineered the natural ϕ29 three-way junction motif to generate an alternative ‘bio-orthogonal’ structure (named F30) that enabled stable aptamer expression in cells. RNAs tagged with this structure showed substantially increased stability compared with RNAs tagged with tRNA scaffold that were targeted for endonucleolytic cleavage in bacterial cells. Here, we considered arm 1 of the engineered ‘bio-orthogonal’ F30-Broccoli sequence to be a suitable point for spliting-up the fused aptamer: 8 base pairs of F30 sequence were included in our split aptamer sequences (Fig. 2) in order to promote the hybridization of the two split sequences in the presence of the complementary Split1/Split2 DBS. Furthermore, this 8 base pair sequence can act as a spacer between aptamer and DBS scaffold when the RNA origami is synthesized including the split aptamer system as a tag. The terminal 4-nt loop UUCG was removed from the Broccoli aptamer stem and split aptamer sequences were elongated in 5′ or 3′ end with RNA sequences complementary to a pre-selected DBS (complementary Split1/Split2 DBS, Fig. 2).

Recently, Alam *et al*.^46^ reported a different strategy: a split-Broccoli system was designed by inserting two Broccoli monomers into arms 2 and 3 of the three way junction motif from ϕ29 pRNA. In addition several base pairs were appended on the two distal stems to promote the complete hybridization of the two strands and the formation of the desired secondary structure. The three way junction dimeric Broccoli was divided into two autonomous RNA strands (called Top and Bottom), that were able to self-assemble and restore the fluorescence function when hybridizing. In contrast here, the hybridization between Split1 and Split2 without the presence of the complementary Split1/Split2 DBS was undesiderable. Thus, we went a step further, presenting a split system that was able to promote the fluorescence activation only in the presence of a target complementary sequence.

**Figure 3:**
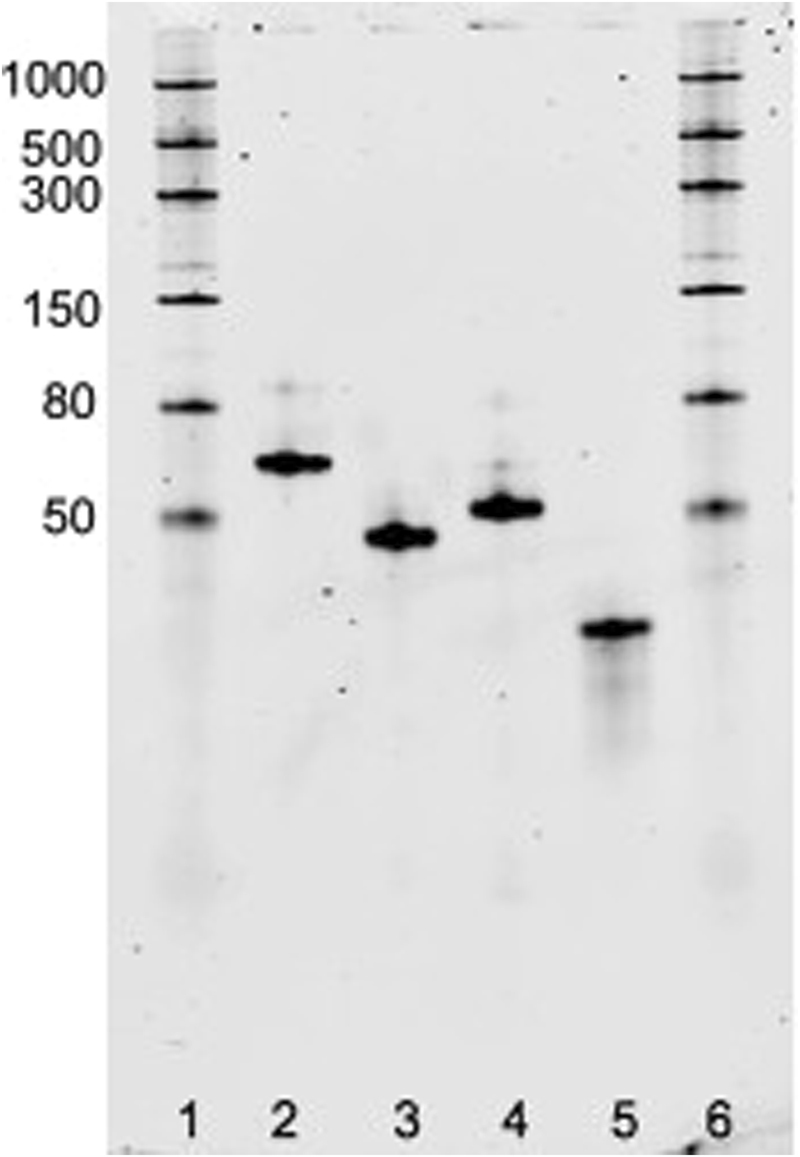
10% TBE-Urea gel electrophoresis after SYBR® Gold staining. Lanes: 1, 6: ssRNA ladder; 2: Broccoli aptamer; 3: Split1; 4: Split2; 5: complementary Split1/Split2 De Bruijn sequence. Molecular sizes in nucleotides are indicated.

**Figure 4:**
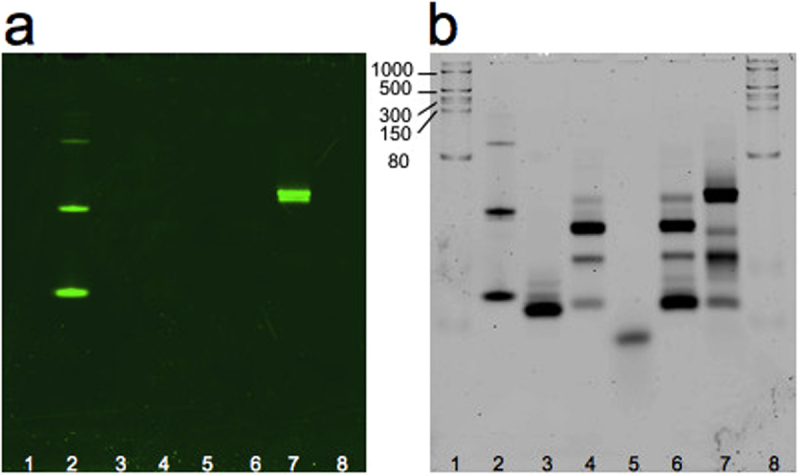
10% TBE gel electrophoresis after DFHBI-1T (**a**) and SYBR® Gold (**b**) staining. The gel was stained with fluorophore DFHBI-1T for 20 min to visualize the Broccoli aptamer and the assembly of Split1, Split2 and complementary Split1/Split2 De Bruijn sequence. After 3 washing steps, the gel was stained with SYBR® Gold for 10 min to detect nucleic acids. Lanes: 1, 8: ssRNA ladder; 2: Broccoli aptamer; 3: Split1; 4: Split2; 5: complementary Split1/Split2 De Bruijn sequence; 6: Split1 and Split2 after incubation at 37 °C for 25 min; 7: Split1, Split2 and complementary Split1/Split2 De Bruijn sequence after incubation at 37 °C for 25 min. Molecular sizes in nucleotides are indicated.

After polymerase chain reaction, the obtained dsDNA with T7 promoter were used as template for the *in vitro* transcription: the transcribed products were analyzed by denaturing gel that confirmed the expected size (Fig. 3). To test the assembly between Split1, Split2 and complementary Split1/Split2 DBS and the fluorescence activation, the *in vitro* transcribed RNAs were incubated at 37 °C for 25 min and resolved by native polyacrilamide gel electrophoresis (PAGE). After washing steps, the gel was stained with DFHBI-1T which binds to Broccoli with no background fluorescence^45^. Fluorescent imaging of the gel revealed bright bands corresponding to Broccoli aptamer and Split1, Split2, complementary Split1/Split2 DBS assembly, thus demonstrating that the fluorescence was restored only after the assembly process of the new split aptamer system (Fig. 4a). On the other hand, each split sequence and complementary DBS alone migrated as a non fluorescent band. Next, the gel was washed and stained with SYBR® Gold to non specifically stain nucleic acids (Fig. 4b). The double staining with different dyes has thus been successfully used to rapidly test the new split aptamer system in a simple way.

**Figure 5:**
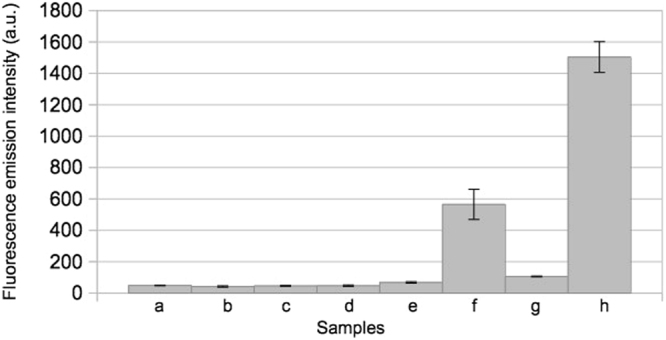
Fluorescence emission intensity (expressed in arbitrary units, a.u.) in 40 mM HEPES pH 7.4, 100 mM KCl, 1 mM MgCl_2_. From left to right: (**a**) background; (**b**) 1 μM Split1; (**c**) 1 μM Split2; (**d**) 1 μM complementary Split1/Split2 DBS; (**e**) 0.5 μM Split1 and 0.5 μM Split2; (**f**) 0.5 μM Split1, 0.5 μM Split2 and 0.5 μM complementary Split1/Split2 DBS; (**g**) 1 μM Split1 and 1 μM Split2; (**h**) 1 μM Split1, 1 μM Split2 and 1 μM complementary Split1/Split2 DBS (mean ± standard deviation, n = 3). All hybridizations were performed at 37 °C for 25 min.

**Figure 6:**
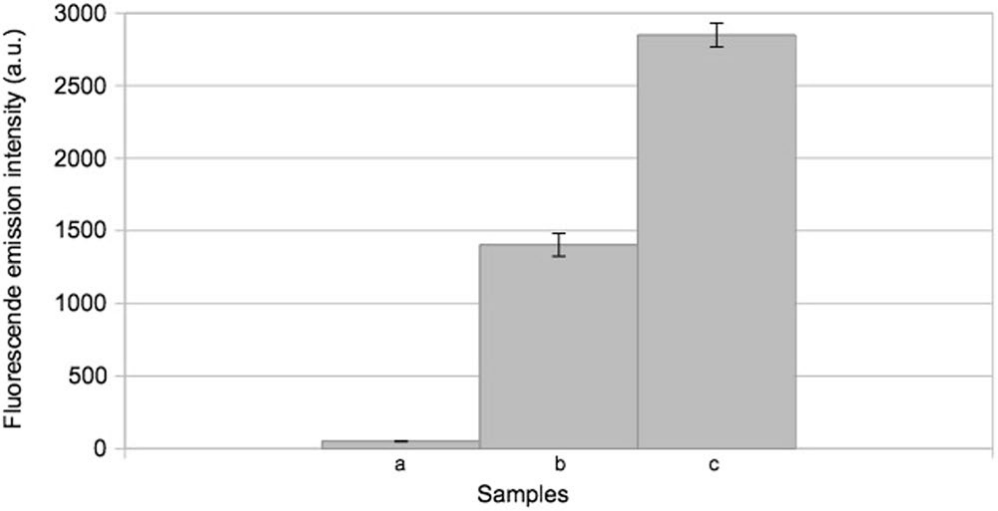
Fluorescence emission intensity (expressed in arbitrary units, a.u.) in 40 mM HEPES pH 7.4, 100 mM KCl, 1 mM MgCl_2_. From left to right: (**a**) background; (**b**) 0.5 μM Broccoli aptamer; 1 μM Broccoli aptamer (mean ± standard deviation, n = 3).

In order to further demonstrate the behaviour of our split aptamer system, Split1, Split2 and complementary Split1/Split2 DBS were mixed and, after incubation at 37 °C for 25 min, the fluorescence intensity was measured. The limit of detection corresponding to a light emission intensity value of 54 arbitrary units (a.u.) was calculated based on the standard deviations of the blank (10 independent measurements). The fluorescence emission values of Split1, Split2, complementary Split1/Split2 DBS alone and Split1 plus Split2 were comparable to the background value, as reported in Fig. 5. The addition of equimolar quantity of complementary Split1/Split2 DBS to Split1 and Split2 showed assembly of the split aptamer system, indicated by higher fluorescence emission intensity values. The fluorescence of the assembled samples at 0.5 µM or at 1 µM were 40% and 53% respectively of the signal from Broccoli aptamer (Fig. 6). These data clearly demonstrate that our split system restored the fluorescence after the addition of a complementary sequence as already demonstrated by in-gel imaging. Our split aptamer system can be used to monitor specific RNA sequences instead of complementary Split1/Split2 DBS. Split aptamer can be elongated in 5′ or 3′ end with RNA sequences complementary to a pre-selected target RNA, thus offering a new tool for applications based on RNA-RNA interactions (e.g. RNA based sensors).

We next performed fluorescence emission intensity measurements on Split1_Split2_complementary Split1/Split2 DBS assembly reducing the magnesium chloride concentration from 1 mM to 0.5 mM, a concentration value compatible to the amount of cellular free magnesium. After incubation in a buffer containing 0.5 mM or 1 mM MgCl_2_, Split1_Split2_complementary Split1/Split2 DBS showed comparable fluorescence emission intensity values (see Supplementary Fig. S2). This result is consistent with a comparison of fluorescence levels measured in buffer containing different magnesium concentrations: Broccoli showed markedly reduced magnesium dependence compared to other aptamers like Spinach and Spinach2^31^ in which divalent ions enhanced fluorescence activation in the presence of monovalent cations^47^.

Finally prior to measuring the fluorescence emission intensity of Split aptamer sequences in the context of the RNA origami, a comparative study of the fluorescence value in the aptamer buffer and in the folding buffer with different K^+^ concentration was performed. Reducing the K^+^ concentration from 100 mM to 0 mM clearly influenced the emission intensity. At the same concentration, the level of fluorescence in aptamer buffer was comparable to that measured in folding buffer. On the other hand, when K^+^ concentration was reduced in both buffers, the fluorescence decreased accordingly (see Supplementary Fig. S3). These results indicated that the folding buffer supplemented with 100 mM KCl is suitable for fluorescence measurements on the folded RNA origami. Recently, it has been revealed from crystal structure of Spinach-DFHBI complex that K^+^ are part of the folded DFHBI-Spinach complex structure. Potassium ions stabilize G-quadruplex structure activating the fluorescence^47,48^: the ability to enhance fluorescence is related to the ability of this cation to support quadruplex formation^49^. As Broccoli retains most of the residues of the G-quadruplex DFHBI binding pocket of Spinach2^31^, here we postulated that K^+^ can play a stabilizing role like in Spinach aptamer.

Taking into account the no toxicity of DFHBI-1T, the ability to assemble at 37 °C without initial thermal denaturation and the reduced magnesium dependance, our light-up split aptamer can be considered as a potential tool for protein-free fluorescence detection of endogenous RNA in live bacterial cells.

### RNA origami: synthesis and atomic force microscopy imaging

Several RNA nanostructures have been synthesized using mainly the self-assembly of natural building blocks. Despite the DNA origami technique being introduced in 2006 by Rothemund^6^, only recently RNA origami nanostructures have been synthesized and well characterized by atomic force microscopy^13^, thus promoting the origami technique in the RNA nanotechnology field.

DNA origamis were usually synthesized by a temperature gradient with an initial denaturation step. DNA staple strands were mixed with DNA scaffold, heated at high temperature and annealed by slow and stepwise cooling to reach room temperature within hours^6^. Using a thermal gradient, Endo and collaborators^13^ synthesized RNA origamis by annealing RNA staple strands and scaffold from 75 to 20 °C at a rate of −0.5 °C/min.

In addition to gradient-based assembly, Sobczak and coll^50^ demonstrated that the hybridization of staple strands, called folding, can occur at a specific and constant temperature. After a brief initial thermal denaturation step, DNA staple strands cooperatively folded a DNA scaffold at constant temperature^50^. Here we went a step further demonstrating the RNA origami isothermal folding at a physiologically compatible temperature (37 °C). Detail of its design is provided as supplementary information (see Supplementary Fig. S4).

After an initial thermal denaturation (75 °C for 1 min) and a snap cooling step (−1 °C/0.42 sec) from 75 to 37 °C, RNA origami were folded in isothermal condition at 37 °C for 20 min. Quality control of RNA origami was done by gel electrophoresis and atomic force microscopy. The quality of the gel bands provides a preliminary check about the quality of the folding and if smeared bands appeared rather than distinct bands, nanostructures are not folded^51^. Here, gel electrophoresis assay of the reaction product showed a distinct band suggesting the well-folded nanostructure formation (see Supplementary Fig. S5). On the other hand, smeared bands were obtained with samples heated at 75 °C or heated at 75 °C and snap cooled at 37 °C (see Supplementary Fig. S6).

**Figure 7:**
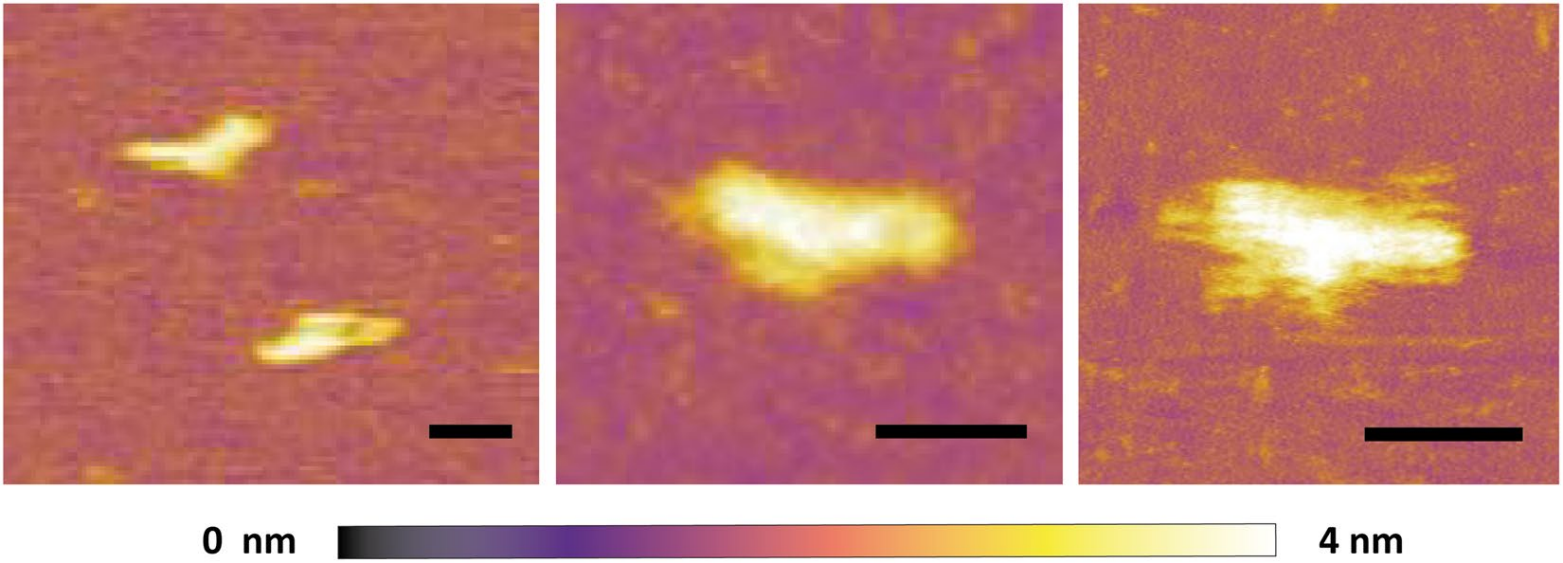
Detailed AFM images of RNA origami using a Cypher ES AFM, Asylum Research equipped with direct laser excitation (blueDrive); scale bar 20 nm.

After folding, the nanostructures were purified using centrifugal filter in order to remove the staple strand contamination, and visualized by AFM. Studies on double stranded RNA in its A-form revealed a rise per base pair of 0.28 nm^52^, thus the estimated dimensions of our RNA origami were approx. 27 nm × 5 nm. AFM images confirmed the correct formation of the RNA origami: their average lenghts were 25.6 nm ± 2.7 nm and 5.3 nm ± 0.8 nm in accordance with the expected size (Fig. 7, see Supplementary Figs S7, S8, S9 and S10). RNA nanostructures interactions were imaged by AFM, presumably due to interaction between left and right scaffold short side. Indeed in our design (see Supplementary Fig. S4), the RNA scaffold contains a left short side (5′-UGUUAUACAG-3′) and a right short side (5′-CCGGUCCGGC-3′), both of them non complementary to any staple strands thus remaining as ssRNA. As previously reported^23,53^, RNA helices can be produced using two to six nucleotides of RNA and only two G/C pairs were also able to provide assembly activity.

Finally, the fraction of purified RNA origami appearing as well-folded nanoribbon was 74% (N = 244). This AFM yield was determined as a percentage of identifiable shapes in an entire AFM field, as previously described^54^. As smaller nanostructures could result from purification sample damage and fragmentation during sample deposition and AFM imaging, the AFM yield percentage could be an understimated value^54^.

### Light-up RNA origami: fluorescence measurements and in-gel imaging

Recently, an alternative approach to the RNA origami method was provided: a single-stranded RNA origami was designed to fold during RNA transcription and a RNA aptamer-based FRET (apta-FRET) system was introduced in the nanostructure. In detail, authors showed FRET between fluorescent RNA aptamers by placing Spinach and Mango in close proximity on a single-stranded RNA origami 2H-AE 2 helix construct^55^. Due to the 2H-AE construct dimension, AFM characterization was performed on larger lattices obtained from 2H-AE assembly^30^ and the synthesis of the apta-FRET nanostructures was demonstrated by recording FRET outputs based on spectrofluorometric and flow cytometry measurements^55^.

On the other hand, here we present a different strategy. Compared to the single stranded RNA nanostructures reported in 2018^55^, our RNA origami utilized seven staple strands that hybridized with a ‘bio-orthogonal’ ssRNA scaffold folding it into a nanoribbon well characterized by AFM. The Broccoli split aptamer system was inserted in the ‘bio-orthogonal’ RNA origami nanoribbon (see Supplementary Fig. S4). The complementary Split1/Split2 DBS was included as part of the DBS used as scaffold for the RNA origami self-assembly. Split1 and Split2 were converted into two staple strands, called Staple s1 and Staple s2 respectively (see Supplementary Table S1), lacking T7 promoter. To demonstrate the fluorescence activation due to the incorporated split aptamer system and to confirm the self-assembly, we used fluorescence measurements and selective in-gel imaging. Furthermore, two subsets of staples were used to partially fold the ‘bio-orthogonal’ scaffold: i) Staple s1 and Staple s2 (sample Sc 2St) and ii) Staple s1, Staple s2, Staple l1, Staple r1 (sample Sc 4St). The Sc 2St and Sc 4St reaction products were run on native PAGE: Sc 2St and Sc 4St samples showed a faster smeared band, while RNA origami sample produced an higher band (see Supplementary Fig. S11).

After RNA origami synthesis, fluorescence measurements on purified RNA nanostructures samples showed a normalized fluorescence value of 0.7 (standard deviation: ±0.1, n = 3). The normalized fluorescence value of the Sc 2St sample was 0.1 (standard deviation: ±0.03, n = 3). No difference was recorded between Sc 4St sample and background. These data showed the fluorescence activation due to the incorporation of the split aptamer system and to the formation of the active Broccoli aptamer on the RNA origami. The RNA origami folding was succefully confirm by fluorescence measurements. Moreover considering the purified RNA origami distinct band on native PAGE and the high folding yield (74%), the fluorescence value could be mainly determined by well-folded nanostructures.

**Figure 8:**
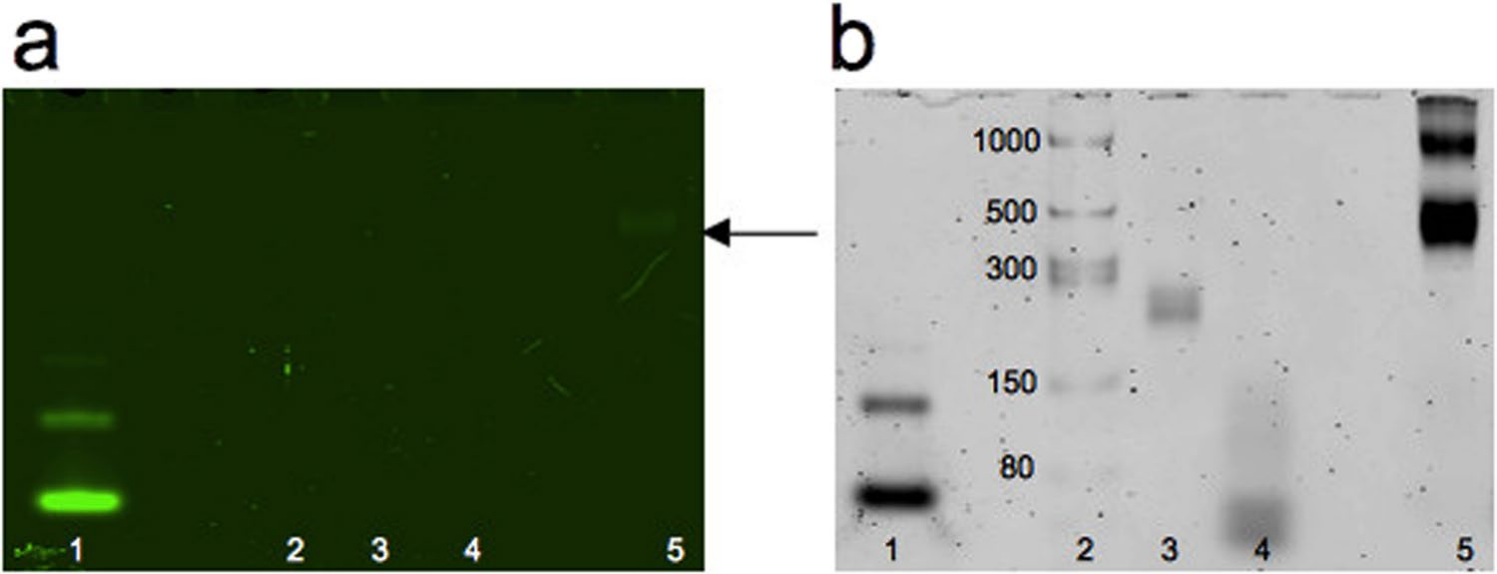
6% TBE gel electrophoresis of Broccoli aptamer (positive control) and RNA origami after DFHBI-1T (**a**) and SYBR® Gold (**b**) staining. The gel was stained with fluorophore DFHBI-1T for 20 min to visualize both Broccoli aptamer and RNA origami. RNA origami was able to bring Split1 and Split 2 into close proximity to restore the fluorescence in the presence of DFHBI-1T. After 3 washing steps, the gel was stained with SYBR® Gold for 10 min to detect nucleic acids. Lanes: 1: Broccoli; 2: ssRNA ladder; 3: RNA scaffold; 4: RNA staples; 5: RNA origami. The black arrow shows the fluorescent RNA origami. Molecular sizes in nucleotides are indicated. Full-length gels are presented in Supplementary Fig. S10.

In order to further confirm the RNA origami folding, light-up RNA nanoribbons were analysed by selective in-gel imaging. Filonov *et al*.^45^ developed an in-gel staining system as a Northern blotting alternative for the detection of RNA cleavage products tagged with Broccoli aptamer. Different molecular weight bands were separated by native PAGE: RNAs that contained Broccoli tag were visualized based on specific binding of DFHBI-1T to the aptamer tag. In our work, in-gel staining confirmed the RNA origami folding: RNA nanostructures migrated as a distinct band and triggered the fluorescence emission in the presence of the specific fluorophore (Fig. 8a, see Supplementary Fig. S12a for full lenght gel). After staining with SYBR® Gold, the bands analysis confirmed the correct migration distance of the RNA origami (Fig. 8b, see Supplementary Fig. S12b for full length gel).

Both different migration pattern and lower normalized fluorescence intensity characterized partially folded samples. This result suggested that fluorescence measurements and in-gel imaging techniques allowed the rapid and selective monitoring of the RNA origami self-assembly through the split aptamer system.

### Labeled scaffold electroporation in *E. coli*

Recently, the influence of electronic pulse on magnesium folded DNA origami structures has been investigated. Magnesium nanostructures disintegrated in the electric fields, as demonstrated by transmission electron microscopy and agarose gel electrophoresis. The electrical pulse moved away the stabilizing magnesium ions: DNA origami was destroyed by the electrostatic repulsion^56^. On the basis of this result, we decided to evaluate the ‘bio-orthogonality’ of the single stranded RNA scaffold electroporated in *E. coli* cells.

Electrocompetent *E. coli* cells were subjected to high-voltage electric field in the presence of Alexa 488 labeled ‘bio-orthogonal’ and no ‘bio-orthogonal’ scaffolds (see Supplementary Figs S13 and S14). Before washing steps and lysis, the cell suspension was recovered for 25 min. Cell-based fluorescence measurements showed that after 30 min the cell division started: the fluorescence was distributed to daughter cells with a fluorescence intensity percentage reduction ranging from 10 to 30%^57^. Considering this fluorescence reduction starting from 30 min, we decided to apply a recovery time of 25 min which was long enough to expect RNA degradation. Indeed genome-wide RNA degradation study in *E. coli* using RNA-seq has been reported and the RNA average life time was 2.5 min. This life time was generally constant across RNAs^58^. For this reason, the RNA turnover machinery is a target of phage effector proteins in order to avoid the rapid exogenous RNA degradation^59–61^.

After the recovery period the excess of non internalized molecules was removed by washing with PBS, as previously described^57^. The cell suspension was embedded in agarose plugs, lysed and loaded into agarose gel wells. To assess the resistance of electroporated scaffold inside the cells, samples were run in agarose gel and analysed: DBS scaffold showed a band of the expected size, while no electroporated *E. coli* showed no bands (see Supplementary Figs S15 and S16). Two different no ‘bio-orthogonal’ RNA sequences (noDBS1 and noDBS2; Table S1) were used as negative control: both sequences didn’t show a distinct band (see Supplementary Figs S17 and S18). This result is consistent with our previous data^8^, thus confirming the ‘bio-orthogonality’ of the scaffold sequence designed *in silico*.

Finally, recent studies showed a broad distribution of electroporated molecules: Crawford *et al*.^62^ reported a mean number of internalized dsDNA. As the band intensity variation can be correlated to a different number of internalized RNA molecules, different recovery periods were not tested to evaluate the band intensity over the time.

## Conclusion

In this study, we designed and characterized a RNA origami. We demonstrated the use of our split Broccoli system as a tag to monitor the isothermal folding at physiological temperature of RNA nanostructures using a cell compatible dye.

Our Split aptamer system assembled at physiological temperature (37 °C) without initial denaturation and at lower magnesium concentration, which could result in future *in vivo* applications. Furthermore, the split Broccoli system restored the fluorescence only in the presence of a specific RNA target sequence, the complementary Split1/Split2 DBS, suggesting the design of a new RNA-based sensor for the detection of RNA hybridization events.

To the best of our knowledge, we are the first to investigate and combine three important aspects: i) ‘bio-orthogonality’; ii) isothermal folding at 37 °C of a nanostructure by employing scaffolded origami strategy instead of a single stranded origami approach and iii) self-assembly monitoring using a new split Broccoli aptamer system. Both unimolecular folding and scaffolded origami represent different approach fo synthesizing nanostructures^63^. Compared to the single stranded origami strategy based on a self-folding^55^, we demonstrated the isothermal self assembly at 37 °C of a ‘bio-orthogonal’ scaffold using multiple staple strands. Finally, the development of a new split Broccoli aptamer system allowed us to confirm the self-assembly.

Our approach can find new development for *in vivo* applications without the introduction of the burden related to fluorescence protein complementation^64^ commonly used to detect RNA assembly^11^ or to label native RNA^65^ *in vivo*. In the perspective of the RNA origami expression and folding in living cells, we take into account all the above mentioned important characteristics. Natural and engineered RNA can control many aspects of the gene expression inside the cells. These regulatory functions can be tuned by the programmable formation of specific RNA structure^12^. Towards this vision, RNA origami nanostructure can represent a useful platform to organize new genetic control system.

## Methods

### Materials and reagents

(5*Z*)-5[(3,5-Difluoro-4-hydroxyphenyl)methylene]-3,5-dihydro-2-methyl-3-(2,2,2-trifluoroethyl)-4*H*-imidazol-4-one (DFHBI-1T) was purchased from Tocris Bio-techne. A DFHBI-1T stock solution (20 mM) was prepared in DMSO and stored in the dark at −20 °C for a week. All RNA oligonucleotides were purchased from Eurogentec and resuspended in ultrapure water to give stock solutions of 100 μM and stored at −80 °C. All DNA oligonucleotides and gBlocks Gene Fragment were purchased from IDT. The DNA oligonucleotides were resuspended in ultrapure water to give stock solutions of 100 μM and stored at −20 °C. The gBlocks Gene Fragment was resuspended at a final concentration of 10 ng µL^−1^ and stored at −20 °C. EDTA 0.5 M pH 8.0, Ultra Pure^TM^ 10x TBE buffer, Ultra Pure^TM^ 1 M Tris HCl pH 7.5, 10x TAE buffer pH 8.5, Ultra Pure^TM^ distilled water, SYBR® Gold, 6% Novex^TM^ TBE gel and 10% TBE-Urea gel were purchased from Invitrogen, Life Technologies. HEPES 1 M pH 7 Bioreagent, KCl 1 M BioUltra, MgCl_2_ 1 M in water BioUltra, 3-aminopropyltriethoxysilane (APTES) and agarose were purchased from Sigma-Aldrich.

### Scaffold design

The synthetic RNA scaffolds were generated with the computer code presented by Kozyra *et al*.^8^. No ‘bio-orthogonal’ scaffolds (noDBS1 and noDBS2) were also considered (see Table S1).

The RNA three-way junction and RNA origami ribbon (see Supplementary Figs S1 and S4) have been designed using caDNAno software^66^.

The formation of three-way junction was simulated with oxRNA coarse-grained model by Šulc *et al*.^67^. Briefly, the system was initialized with three RNA strands: scaffold DBS, Split1 and Split2. The two aptamer parts were pulled towards the corresponding scaffold domains using mutual traps. After the initial hybridisation with the scaffold the system was simulated for 4.64 ms at 37 °C and the formation of aptamer was observed.

### Synthesis of Broccoli aptamer, Split1 and Split2 transcript sequences

The dsDNA templates containing T7 promoter sequence were prepared by polymerase chain reaction with Broccoli ssDNA, Split1, Split2, (see Supplementary Table S1), primers (Broccoli forward and reverse, Split 1 forward and reverse, Split 2 forward and reverse; see Supplementary Table S1) and Phusion® DNA polymerase (NEB). An initial denaturation at 98 °C for 30 sec was followed by denaturation at 98 °C for 10 sec, annealing for 20 sec and extension at 72 °C for 15 sec. Finally, an additional extension was achieved for 5 min at 72 °C. Annealing temperatures and number of cycles for each template are listed in Supplementary Table S2. The size of amplicons were evaluated on 1% agarose gel in Tris Acetate EDTA (TAE) for 1 h at 100 V: gel was stained for 10 min in ethidium bromide (0.5 µg mL^−1^) and visualized under UV illumination. The low range molecular weight DNA ladder (NEB) was used as molecular weight marker. The PCR products were purified using Monarch® PCR & DNA Cleanup kit (NEB) and DNA concentration was measured on a NanoDrop spectrophotometer. Ampliscribe^TM^ T7-Flash^TM^ Transcription kit (Epicentre) was used to transcribe single stranded RNA from DNA template: a total of 20 µL mixture was prepared and incubated at 37 °C for 1 h and 30 min. After DNase treatment at 37 °C for 15 min, the RNA transcripts were purifed using RNA Clean & Concentrator^TM^ (Zymo Research) and quantified using a NanoDrop spectrophotometer. After the addition of the RNA loading dye 2x (NEB) and the heat denaturation at 65 °C for 5 min, *in vitro*-transcribed RNAs were loaded and run on 10% TBE-Urea gel in 1x Tris borate EDTA (TBE) buffer at 200 V for 40 min. After staining with SYBR® Gold in 1x TBE for 8 min, the gels were visualized using Typhoon laser scanner and Image Quant TL software (normal sensitivity and PMT 500 or 600 V; GE Healthcare Life Sciences). The low range ssRNA ladder (NEB) was used as molecular weight marker.

### Assembly of Split sequences with **co**mplementary DBS and in-gel imaging

Split 1 RNA, Split 2 RNA and complementary Split 1/Split 2 DBS were mixed in a 1:1:1 ratio and incubated together at 37 °C for 25 min. All the samples including negative controls (Split sequences without complementary DBS) were run on 6% Novex^TM^ TBE gel in 1x TBE buffer at 200 V for 45 min. In-gel imaging was performed as previously described^45^. Briefly, the gels were washed three times for 5 min in RNase free water and then stained for 25 min in 10 μM DFHBI-1T in aptamer buffer containing 40 mM HEPES pH 7.4, 100 mM KCl, 1 mM MgCl_2_. The gels were imaged using Typhoon laser scanner (excitation 488 nm, emission 532 nm). Then, the gels were washed three times with RNase free water, stained with SYBR® Gold in 1x TBE for 8 min, and visualized using Typhoon laser scanner. The low range ssRNA ladder (NEB) was used as molecular weight marker.

### Spectrofluorometer measurements on Split aptamer assembly

The fluorescence was measured with ClarioStar Plate Reader (BMG Labtech) and samples were excited at 460 nm and the emission was recorded at 544 nm. Samples and blanks (35 µL each) were loaded on a 96-well RNAse free µClear® black microplate (Greiner Bio-One) according to the following specifications. Negative controls (Split sequences in a 1:1 ratio at a final concentration of 0.5 μM or 1 μM, without complementary DBS), Split1 RNA_Split2 RNA_complementary Split1/Split2 DBS were mixed in a 1:1:1 ratio (at a final concentration of 0.5 μM or 1 μM) and incubated at 37 °C for 25 min, as reported above. After the incubation period at 37 °C, the aptamer buffer was added to reach a final volume of 34.55 µL. The fluorophore solution (0.45 µL from a 0.8 mM DFHBI-1T in DMSO stock solution) was added to all samples and fluorescence intensities were recorded.

### RNA scaffold synthesis and purification

Double stranded gBlocks Gene Fragment containing T7 promoter (gBlocks DBS scaffold, see Supplementary Table S1) was amplified using Phusion® DNA polymerase (NEB) and DBS forward/DBS reverse primers (see Supplementary Table S1). An initial denaturation at 98 °C for 30 sec was followed by 15 cycles of denaturation at 98 °C for 10 sec, annealing at 60 °C for 20 sec and extension at 72 °C for 15 sec. Finally, an additional extension was achieved for 5 min at 72 °C. The size of amplicon was evaluated on 1% agarose gel in TAE for 1 h at 100 V: gel was stained for 10 min in ethidium bromide (0.5 µg mL^−1^) and visualized under UV illumination. The low range molecular weight DNA ladder (NEB) was used as molecular weight marker.

The PCR product was purified using Monarch® PCR & DNA Cleanup kit (NEB) and the DNA concentration was measured on a NanoDrop spectrophotometer. The purified template was transcribed *in vitro* at 37 °C for 1 h and 30 min using Ampliscribe^TM^ T7-Flash^TM^ Transcription kit (Epicentre). The noDBS1 and noDBS2 scaffold sequences were transcribed *in vitro* at 37 °C for 2 h. After DNase treatment at 37 °C for 15 min, the RNA transcript was purifed using RNA Clean & Concentrator^TM^ (Zymo Research) and quantified using a NanoDrop spectrophotometer. After the addition of the RNA loading dye 2x (NEB) and the heat denaturation at 65 °C for 5 min, *in vitro*-transcribed RNA was loaded and run on 10% TBE-Urea gel in 1x TBE buffer at 200 V for 40 min. After staining with SYBR® Gold in 1x TBE for 8 min, the gels were visualized using Typhoon laser scanner. The low range ssRNA ladder (NEB) was used as molecular weight marker.

### RNA origami assembly and purification

The set of staple strands (see Supplementary Table S1) were mixed in 10-fold excess with RNA scaffold in 50 μL of folding buffer (10 mM MgCl_2_, 20 mM Tris-HCl pH 7.6, 1 mM EDTA pH 8.0)^13^. After an initial thermal denaturation step at 75 °C for 1 min and a snap cooling (−1 °C/0.42 sec), the mixture was subjected to a folding step at 37 °C for 20 min. To confirm that the folding occured isothermically at 37 °C, samples were incubated at 75 °C for 1 min or subjected to an initial thermal denaturation at 75 °C for 1 min followed by snap cooling (−1 °C/0.42 sec).

In order to evaluate fluorescence emission intensity of partially folded nanostructures, two different staples subsets were considered: i) Staple s1 and Staple s2 (sample Sc 2St) and ii) Staple s1, Staple s2, Staple l1, Staple r1 (sample Sc 4St). The partially folded and folded constructs were purified from staple strands excess using the Amicon Ultra 0.5 mL 50 kDa or 100 kDa centrifugal filters (Millipore, Massachusetts). Capped Amicon Ultra were rinsed with 400 µL of folding buffer and centrifuged at 10000 g for 6 min at 15 °C. After this preliminary washing step, the samples were added to the filter device. Between every centifugation step, the flowthrough was removed and the filter was refilled with 400 μL of folding buffer. To recover the sample, the filter was turned upside down and centrifuged once more at 300 g for 10 min at 15 °C.

### RNA origami visualization and in-gel imaging

Samples were run on 6% Novex^TM^ TBE gel in 1x TBE buffer at 100 V for 40 min at low temperature (below 10 °C). After staining with SYBR® Gold in 1x TBE for 8 min, the gels were visualized using Typhoon laser scanner. The low range ssRNA ladder (NEB) was used as molecular weight marker. To confirm the RNA origami self-assembly by incorporation of Split Broccoli aptamer system, in-gel imaging with fluorophore DFHBI-1T was done as described above.

### RNA origami spectrofluorometer measurements

The fluorescence was measured with ClarioStar Plate Reader (BMG Labtech): samples were excited at 460 nm and the emission was recorded at 544 nm. After the addition of 11.2 µL of KCl 1 M and 1.29 µL of fluorophore stock solution (0.8 mM DFHBI-1T in DMSO), purified RNA origami, purified partially folded samples and blanks (final volume: 112.49 µL) were loaded on a 96-well RNAse free µClear® black microplate (Greiner Bio-One) and the fluorescence intensities were recorded.

### Atomic Force Microscopy imaging

Freshly cleaved mica was passivated for 5 min with 10 μL of 0.1% APTES in water to ensure the adhesion of the negative charged RNA origami structures on the negative mica surface. After three washing with folding buffer, purified RNA origami samples (8 μL) were added to the passivated mica surface and allowed to adsorb for 5 min in a chamber. AFM imaging was performed with a Bruker multimode 8 AFM in Scanasyst mode using Bruker Scanasyst-Fluid+ tip for lower resolution images and with a Cypher ES for higher resolution. All images where acquired with the AFM operated in liquid, in tapping mode and with a setpoint ratio between the free amplitude and imaging amplitude of ~0.7. The Cypher was equipped with laser excitation (blueDrive) and temperature control, enabling ultrastable operation and fully reproducible imaging parameters. Measurements were conducted using cantilevers from a same wafer for better comparability (OMCL RC800-PSA, Olympus, Tokyo, Japan) with a nominal spring constant of 0.76 N/m. Systematic thermal calibration^68,69^ showed variations of less than 10% between cantilevers. The sample temperature control set at 20 ± 0.1 °C for all the experiments. All the images were corrected for tilt (line or plane flattening) and lightly low-pass filtered to remove grainy noise using the WSxM software (Nanotec Electronica, Madrid, Spain)^70^.

Folding yield analysis based on AFM imaging was done as previously described^54^.

### Scaffold labeling with Alexa 488 fluorophore

Labeling of DBS, noDBS1 and noDBS2 scaffolds were performed using Ulysis® Nucleic Acid Labeling kit (Life Technologies) following manufacturer’s protocol. Labeled RNA solution was purified by using Micro Bio-Spin® P-30 spin column (BioRad) and eluted in 10 mM Tris-HCl pH 7.4.

The Alexa 488 scaffold was run on 2% agarose gel at 100 V for 1 h: gel was imaged using Typhoon laser scanner (excitation 488 nm, emission 532 nm). After ethidium bromide staining, gel was again imaged using Typhoon laser scanner.

### Electroporation and plug preparation

Electroporation and plug preparation has been done as previously described^8^. NEB 5-alpha electrocompetent *E. coli* cells (NEB) were diluted 1:1 with sterile milli-Q water in a prechilled tube: 20 μL of cells suspension were used for each electroporation experiment after the addition of 5 μL of labeled scaffold (DBS, noDBS1 or noDBS2). The mixture of competent cells and labeled molecules was immediately transferred into a prechilled electroporation cuvette (0.2 cm gap cuvette, BioRad) and electroporated (Gene Pulser, BioRad; 1.8 kV, 200 Ω, 25 μF). The negative control was prepared incubating cells with the same volume of the same labeled scaffold solution: the cells were not electroporated and were washed as the electroporated samples. After electroporation, cells were allowed to recover for 25 min at 37 °C in pre-warm SOC. Then cells were harvested by centrifugation at 3300 g for 1 min at 4 °C, washed 5 times with 500 μL of 1x phosphate buffered saline solution pH 7.4 (PBS, Chem Cruz) and resuspended in 50 μL of PBS^57,62^. 2.5 μL of proteinase K (20 mg mL^−1^ stock, NEB) and 50 μL of melted SeaKem Gold agarose (Lonza) in TE (10 mM Tris, 1 mM EDTA pH 8.0) were added to the cell suspension and mixed gently. The agarose-cell suspension mixture was dispensed into a well of plug mold (BioRad) and allowed to solidify at 4 °C for 20 min^71^.

### Lysis of cells in plugs

The plugs were incubated in 5 mL of cell lysis buffer (50 mM Tris, 50 mM EDTA pH 8, 1% sarcosine, 0.1 mg mL^−1^ of proteinase k) for 15 min at 54 °C in a water bath^71^. After lysis, the plugs were washed four times (10 min/wash) at room temperature (once with nucleases free water and three times with TE pH 8.0). After washing steps the plug slices were loaded into the wells of 1% TBE agarose gel: the electrophoresis was performed for 1 h at 100 V. The 1 kb DNA ladder (NEB) and the low range ssRNA ladder (NEB) were used as molecular weight markers. The gels were imaged using Typhoon laser scanner (excitation 488 nm, emission 532 nm). After SYBR® Gold staining for 25 min, the gels were scanned again on Typhoon laser scanner.

### Data availability

All data generated and analysed during this study are included in this published article (and its Supplementary Information file).

## Electronic supplementary material

Supplementary Information
