# Supplementary material for: Isothermal folding of a light-up bio-orthogonal RNA origami nanoribbon
Source: Sci Rep. 2018 May 3;8:6989. doi: 10.1038/s41598-018-25270-6 (PMC5934368; doi:10.1038/s41598-018-25270-6)
Supplement: Supplementary file 1 — Supplementary Information [file 41598_2018_25270_MOESM1_ESM.pdf]

# Supplementary Information

## Isothermal folding of a light-up bio-orthogonal RNA origami nanoribbon

Emanuela Torelli,[1,*] Jerzy Wieslaw Kozyra,[1] Jing-Ying Gu,[2] Ulrich Stimming,[2] Luca Piantanida,[3] Kislon Voïtchovsky[3] & Natalio Krasnogor[1,*]

[1]Interdisciplinary Computing and Complex BioSystems (ICOS), Centre for Synthetic Biology and Bioeconomy (CSBB), Centre for Bacterial Cell Biology (CBCB), Newcastle University, Newcastle upon Tyne, NE4 5TG, United Kingdom.
[2]School of Chemistry, Newcastle University, Newcastle upon Tyne, NE1 7RU, United Kingdom
[3]Department of Physics, Durham University, Durham, DH1 3LE, United Kingdom
*natalio.krasnogor@newcastle.ac.uk, emanuela.torelli@newcastle.ac.uk

**Table S1.** DNA and RNA sequences used in this study. The capital letters underlined in green correspond to split Broccoli aptamer sequences.

| Oligo name | Sequence [5'-3'] | Length [bases] |
|---|---|---|
| Broccoli ssDNA (DNA sequence with T7 promoter) | TTCTAATACGACTCACTATAGGTATGTGGGAGACGGTCGGG TCCAGATATTCGTATCTGTCGAGTAGAGTGTGGGCTCCCAC ATAC | 86 |
| Broccoli formard primer | TTCTAATACGACTCACTATAGGTATGTGGGAG | 32 |
| Broccoli reverse primer | GTATGTGGGAGCCCACACTCTAC | 23 |
| Split 1 (DNA sequence with T7 promoter) | TTCTAATACGACTCACTATAGGTCCAAAAGAAGGAACCGTAT GTGGGAGACGGTCGGGTCCAGATA | 66 |
| Split 1 forward primer | TTCTAATACGACTCACTATAGGTCC | 25 |
| Split 1 reverse primer | TATCTGGACCCGACCG | 16 |
| Split 2 (DNA sequence with T7 promoter) | TTCTAATACGACTCACTATAGGTATCTGTCGAGTAGAGTGTG GGCTCCCACATACTGGACGAAACTTCCCC | 71 |
| Split 2 forward primer | TTCTAATACGACTCACTATAGGTATCTG | 28 |
| Split 2 reverse primer | GGGGAAGTTTCGTCCAG | 17 |
| Complementary Split 1/Split 2 DBS | GGGGAAGUUUCGUCCAGGUUCCUUCUUUUGGA | 32 |
| gBlocks DBS scaffold | TTCTAATACGACTCACTATAGGTTCCTTCTTTTGGATCACAG TGGAGCAATATCTCGGGTGGCTAGGTTGTTATACAGCCGCG ATATTTATCGGAAAGTCGAGTGCAAGTACCCGGGAGACCGT AACTGAAACCCATAGAACCTTAAACACGATTCGCTCACGGG GGTCACTTACCGGTCCGGCTCGTGTGCTCTCAGTAAAGATG ATCCGCCGATGGGGAAGTTTCGTCCA | 232 |
| DBS forward | TTCTAATACGACTCACTATAGGTTCC | 26 |
| DBS reverse | TGGACGAAACTTCCCC | 16 |
| gBlocks NoDBS1 scaffold | TTCTAATACGACTCACTATAGACAGTATTTGGTATCTGCGCT CTGCTGAAGCCAGTTACCTTCGGAAAAAGAGTTGGTAGCTC TTGATCCGGCAAACAAACCACCGCTGGTAGCGGTGGTTTTT TTGTTGGTTCCTTCTTTTGGATCACAGTGGAGCAATATCTCG GGTGGCTAGGTTGTTATACAGCCGCGATATTTATCGGAAAG TCGAGTGCAAGTACCCGGGAGACCGTAA | 235 |

| | | |
|---|---|---|
| gBlocks NoDBS2 scaffold | TTCTAATACGACTCACTATAGACAGTATTTGGTATCTGCGCT CTGCTGAAGCCAGTTACCTTCGGAAAAAGAGTTGGTAGCTC TTGATCCGGCAAACAAACCACCGCTGGTAGCGGTGGTTTTT TTGTTCTGAAACCCATAGAACCTTAAACACGATTCGCTCACG GGGGTCACTTACCGGTCCGGCTCGTGTGCTCTCAGTAAAG ATGATCCGCCGATGGGGAAGTTTCGTCCA | 235 |
| Staple r1 | CGAGAUAUUGCUCCACUGUGAACUUGCACUCG | 32 |
| Staple r2 | ACUUUCCGAUAAAUAUCGCGGACCUAGCCACC | 32 |
| Staple s1 | UCCAAAAGAAGGAACCGUAUGUGGGAGACGGUCGGGUCC AGAUA | 44 |
| Staple s2 | UAUCUGUCGAGUAGAGUGUGGGCUCCCACAUACUGGACG AAACUUCCCC | 49 |
| Staple l1 | UCGUGUUUAAGAUCGGCGGAUCAUCUUUACUG | 32 |
| Staple l2 | AGAGCACACGAUAAGUGACCCCCGUGAGCGAA | 32 |
| Staple f | GUUCUAUGGGUUUCAGUUACGGUCUCCCGGGU | 32 |

**Table S2.** Annealing temperatures and number of cycles of different templates used during polymerase chain reaction to prepare dsDNA for *in vitro* transcription.

| Template | Annealing temperature [°C] | Number of cycles |
|---|---|---|
| Broccoli ssDNA | 64 | 20 |
| Split 1 | 57 | 25 |
| Split 2 | 57 | 25 |

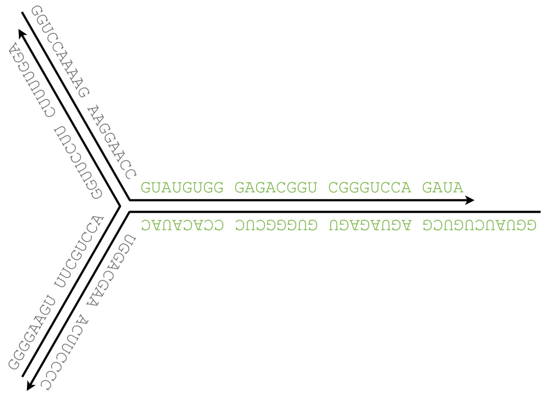


**Figure S1.** The detailed design of the three-way junction. Note the secondary structure of the split aptamer is not shown. The arrowheads indicate 3’ ends.

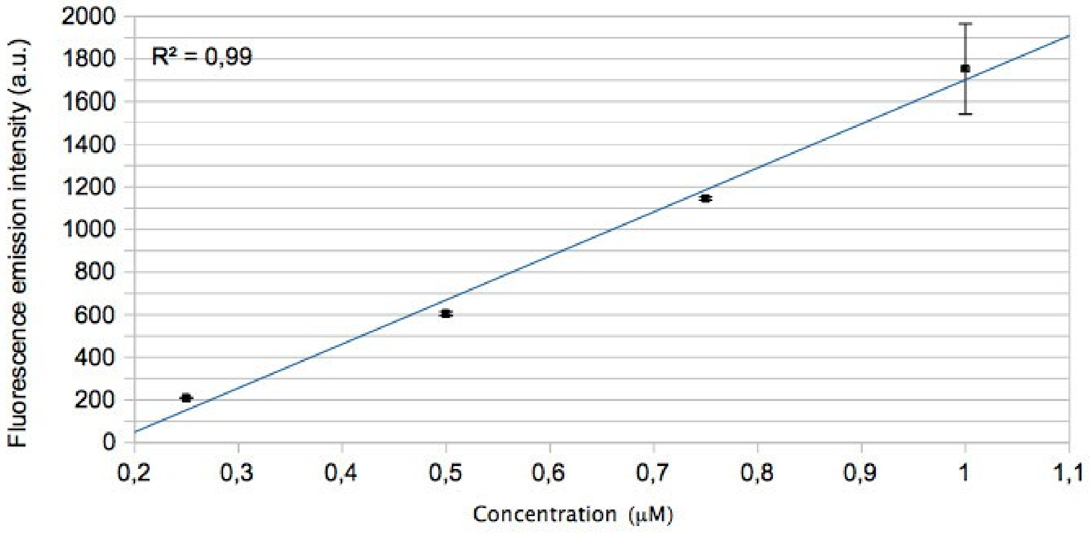


**Figure S2.** Fluorescence emission intensity (expressed in arbitrary units, a.u.) of Split1, Split2 and complementary Split1/Split2 DBS after incubation at 37 °C for 25 min in 40 mM HEPES pH 7.4, 100 mM KCl, 0.5 mM $MgCl_2$. Error bars indicate standard deviation (n=3).

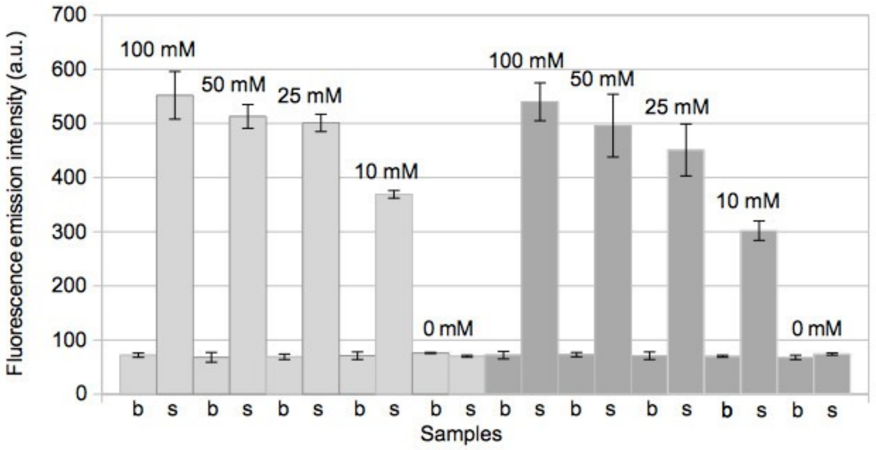


**Figure S3.** Fluorescence emission intensity (expressed in arbitrary units, a.u.) of background and Broccoli aptamer (0.27 μM) in aptamer buffer and in folding buffer with decreasing KCl concentration (100 mM, 50 mM, 25 mM, 10 mM and 0 mM). Aptamer buffer (pale grey): 40 mM HEPES pH 7.4, 1 mM $MgCl_2$. Folding buffer (dark grey): 20 mM Tris HCl pH 7.6, 1 mM EDTA, 10 mM $MgCl_2$. b: background; s: aptamer solution; error bars indicate standard deviation (n=3).

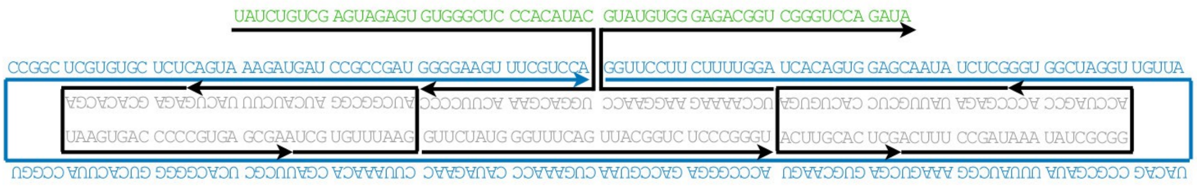


**Figure S4.** The detailed design of the RNA origami ribbon. The arrowheads indicate 3’ ends. The RNA scaffold strand (in blue) is folded with the seven staple strands (in black). Two staples include split Broccoli aptamer sequences (in green).

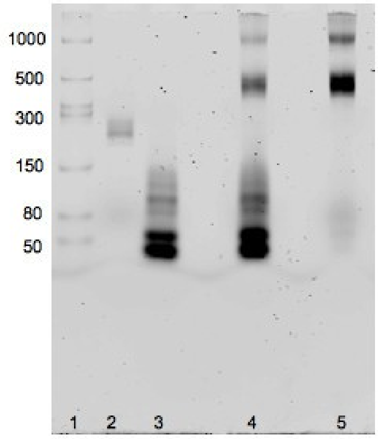


**Figure S5.** 6% TBE gel electrophoresis of well-folded two dimensional RNA origami (initial denaturation at 75 °C for 1 minute followed by RNA assembly at 37 °C for 20 minutes). The assembly reaction of the RNA origami was run and imaged after SYBR® Gold staining. Lanes: 1: ssRNA ladder; 2: RNA scaffold; 3: RNA staples; 4: well-folded RNA origami; 5: purified RNA origami. Molecular sizes in nucleotides are indicated.

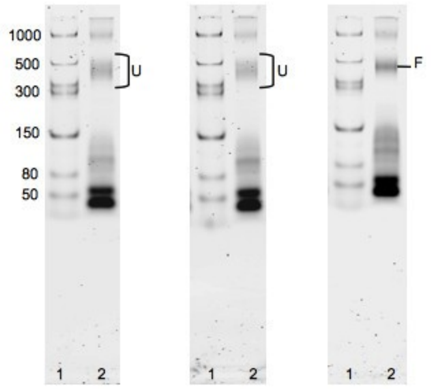


**Figure S6.** 6% TBE gel electrophoresis of unfolded (U) and well-folded (F) two dimensional RNA origami. The unfolded reaction mixtures were obtained by denaturation at 75 °C for 1 min (left image) or by initial denaturation at 75 °C for 1 min and snap cooling (-1°C/0.42 sec; central image). After an initial denaturation step at 75 °C for 1 min, the RNA origami was folded 37 °C for 20 minutes (right image). The reaction mixtures were run and imaged after SYBR® Gold staining. Lanes: 1: ssRNA ladder; 2: unfolded (U) or well-folded (F) RNA origami. Molecular sizes in nucleotides are indicated.

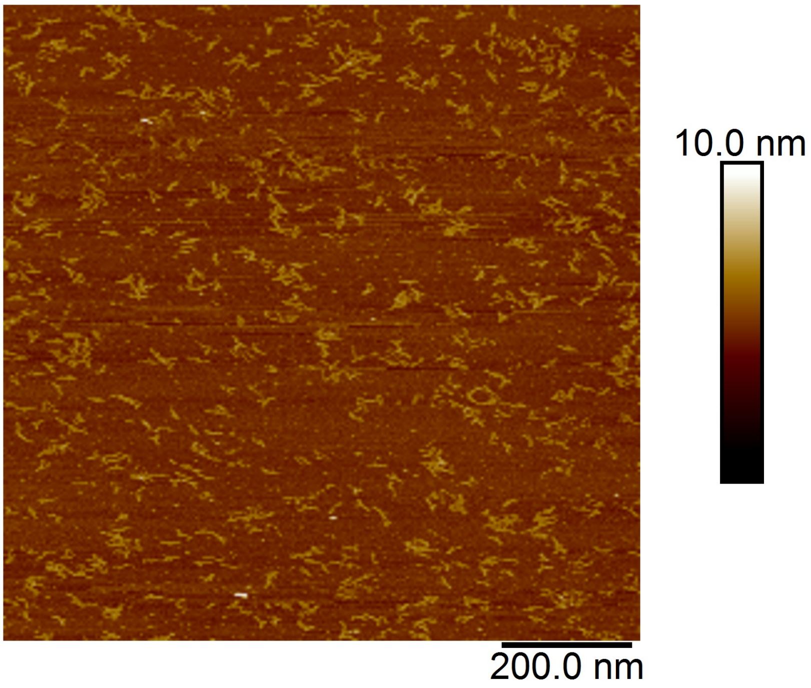


**Figure S7.** Images of RNA origami obtained using a Bruker multimode 8 AFM with Bruker Scanasyst-Fluid+ tip in Scanasyst mode.

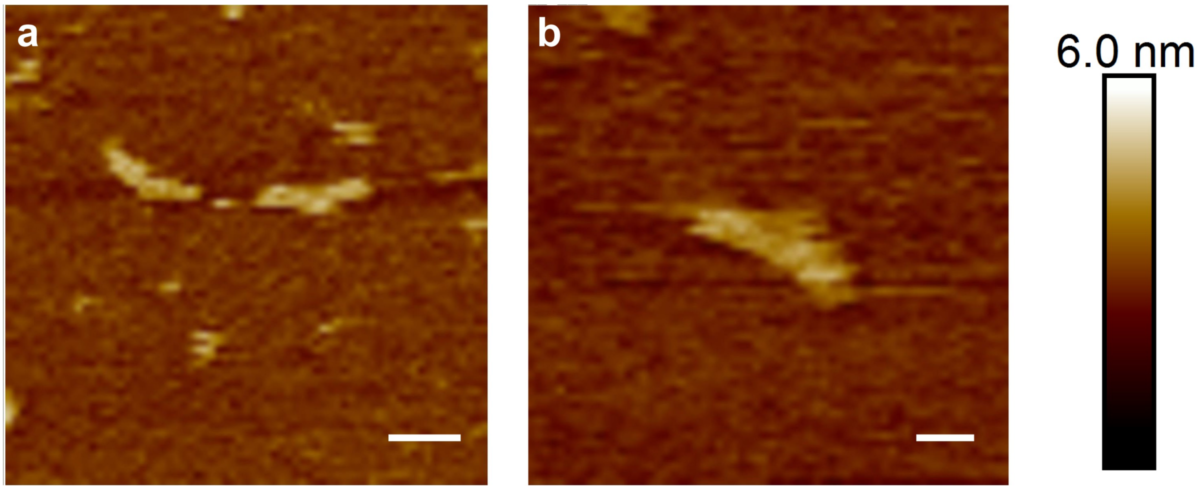


**Figure S8.** Detailed of RNA origami obtained using a Bruker multimode 8 AFM with Bruker Scanasyst-Fluid+ tip in Scanasyst mode (a: scale bar 20 nm; b: scale bar 10 nm).

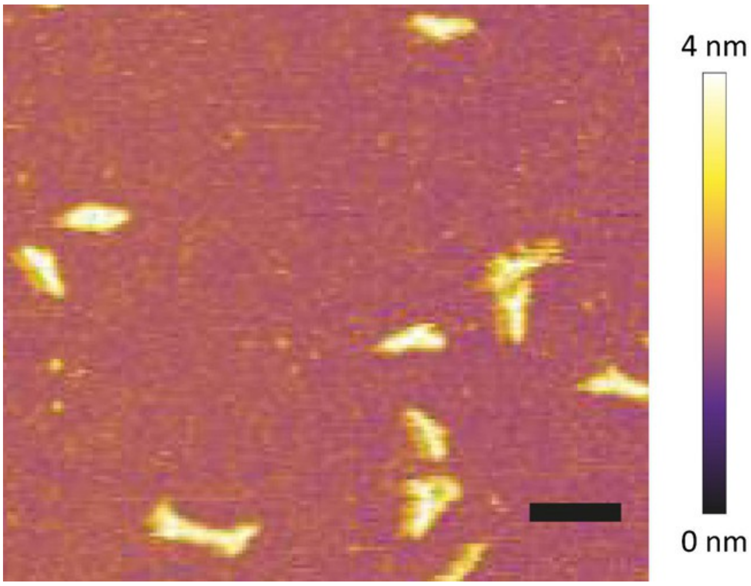


**Figure S9.** Images of RNA origami using a Cypher ES AFM, Asylum Research equipped with direct laser excitation (blueDrive) (scale bar 50 nm).

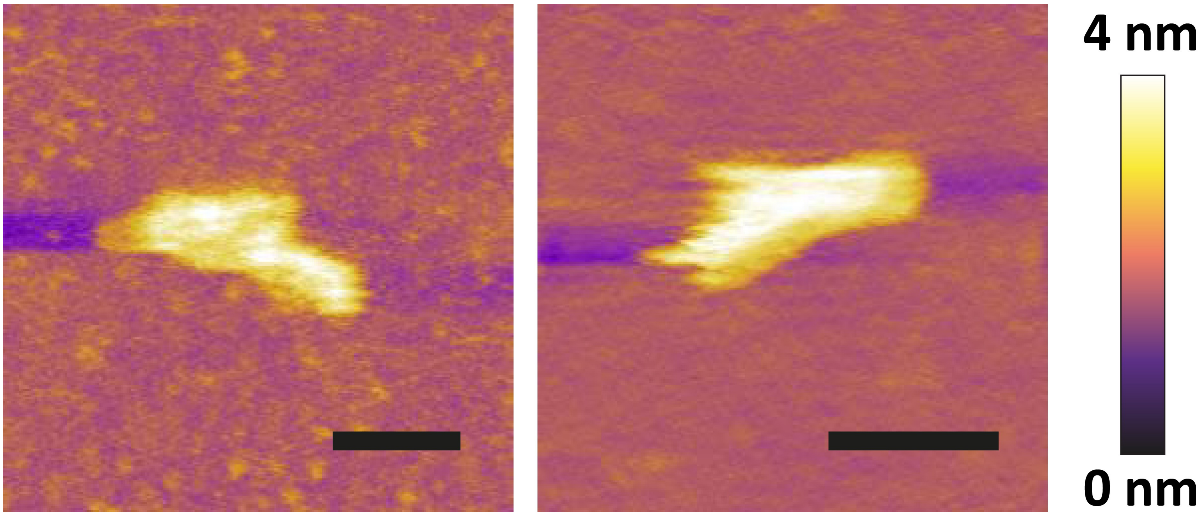


**Figure S10.** Detailed AFM images of RNA origami using a Cypher ES AFM, Asylum Research equipped with direct laser excitation (blueDrive); scale bar 20 nm.

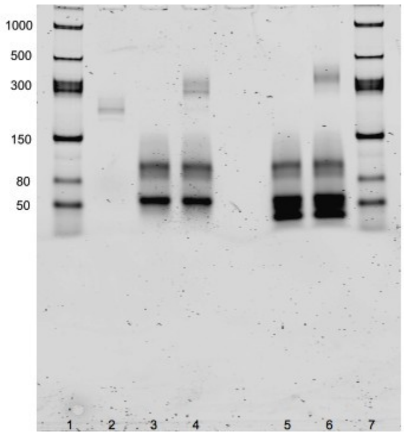


**Figure S11.** 6% TBE gel electrophoresis of partially folded RNA origami. The assembly reactions were run and imaged after SYBR® Gold staining. Lanes: 1: ssRNA ladder; 2: RNA scaffold; 3: RNA Staples s1 and s2; 4: partially folded RNA origami (subset with scaffold, Staple s1 and s2); 5: RNA Staples s1, s2, l1 and r1; 6: partially folded RNA origami (subset with scaffold, Staples s1, s2, l1 and r1). Molecular sizes in nucleotides are indicated.

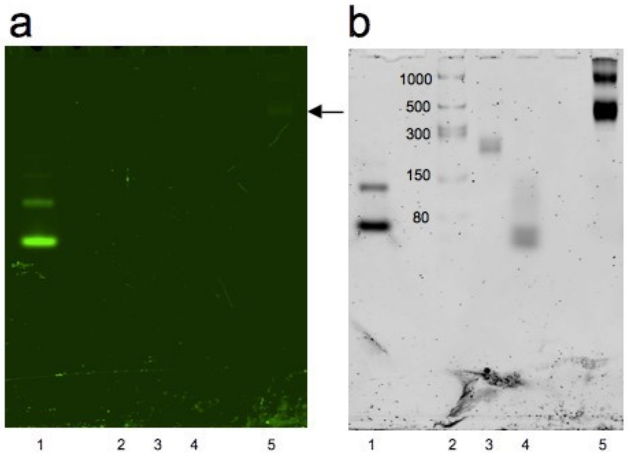


**Figure S12.** 6% TBE gel electrophoresis of Broccoli aptamer (positive control) and RNA origami after DFHBI-1T (a) and SYBR® Gold (b) staining. The gel was stained with fluorophore DFHBI-1T for 20 min to visualize both Broccoli aptamer and RNA origami. RNA origami was able to bring Split1 and Split 2 into close proximity to restore the fluorescence in the presence of DFHBI-1T. After 3 washing steps, the gel was stained with SYBR® Gold for 10 min to detect nucleic acids. Lanes: 1: Broccoli; 2: ssRNA ladder; 3: RNA scaffold; 4: RNA staples; 5: RNA origami. Molecular sizes in nucleotides are indicated. The black arrow shows the fluorescent RNA origami.

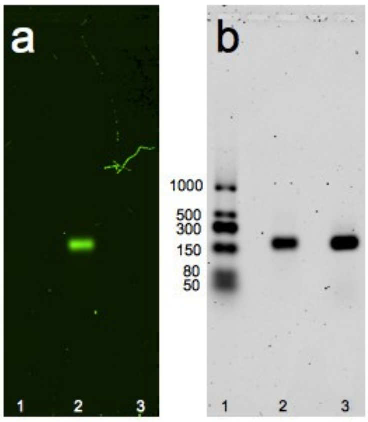


**Figure S13.** Agarose gel electrophoresis of Alexa 488 labeled De Bruijn scaffold sequence. The samples were run on a standard agarose gel and imaged using Typhoon laser scanner before (left) and after (right) SYBR® Gold staining. Lanes. 1: low range ssRNA ladder; 2: Alexa 488 labeled DBS scaffold after purification; 3: not labeled DBS scaffold. Molecular sizes in nucleotides are indicated.

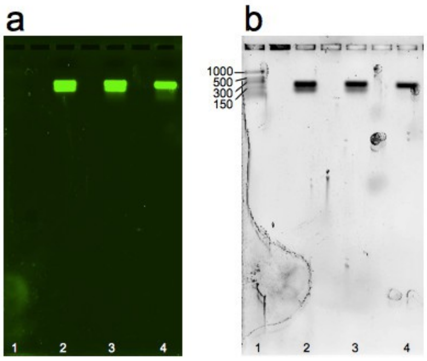


**Figure S14.** Agarose gel electrophoresis of Alexa 488 labeled DBS, noDBS1 and noDBS2 scaffold sequences. The samples were run on a standard agarose gel and imaged using Typhoon laser scanner before (left) and after (right) SYBR® Gold staining. Lanes. 1: low range ssRNA ladder; 2: Alexa 488 labeled noDBS1 scaffold after purification; 3: Alexa 488 labeled noDBS2 scaffold after purification; 4: Alexa 488 labeled DBS scaffold after purification. Molecular sizes in nucleotides are indicated.

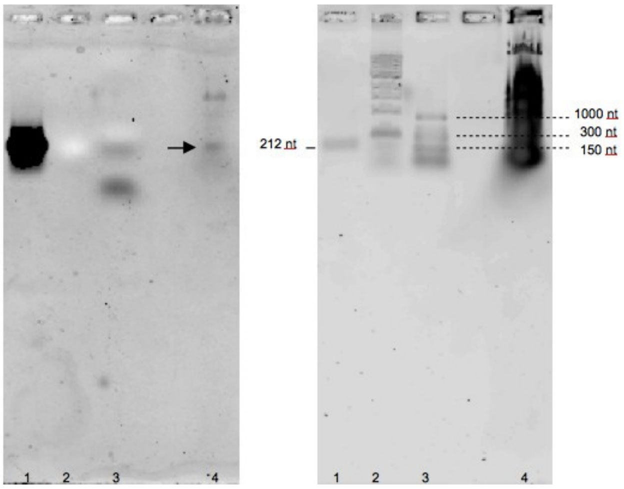


**Figure S15.** Agarose gel electrophoresis of Alexa 488 labeled De Brujin sequence (DBS) electroporated in *E. coli*. Cells were recovered for 25 minutes in SOC medium at 37 °C. After embedding in agarose plugs, *E. coli* cells were lysed, run on a standard agarose gel and imaged using Typhoon laser scanner before (excitation 488 nm; emission 532 nm, left) and after (right) SYBR® Gold staining. Lanes. 1: Alexa 488 scaffold DBS; 2: 1 Kb DNA ladder; 3: ssRNA ladder; 4: electroporated *E. coli* in the presence of Alexa 488 labeled RNA scaffold. Black arrow shows Alexa 488 labeled scaffold.

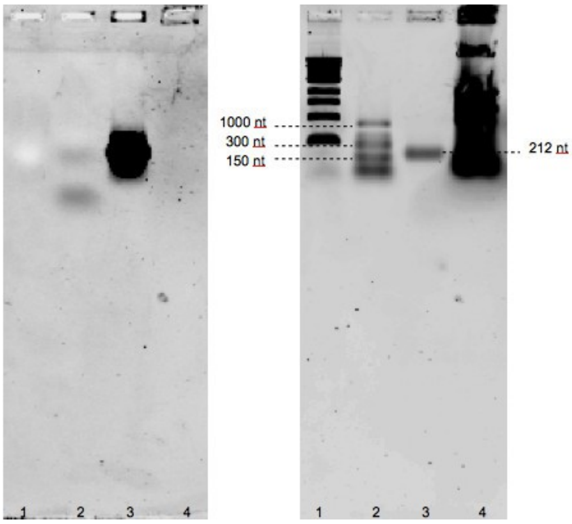


**Figure S16.** Agarose gel electrophoresis of Alexa 488 labeled De Brujin sequence (DBS) not electroporated in *E. coli*. Cells were incubated for 25 minutes in SOC medium at 37 °C. After embedding in agarose plugs, *E. coli* cells were lysed, run on a standard agarose gel and imaged using Typhoon laser scanner before (excitation 488 nm; emission 532 nm, left) and after (right) SYBR® Gold staining. Lanes. 1: 1 Kb DNA ladder; 2: ssRNA ladder; 3: Alexa 488 scaffold DBS; 4: not electroporated *E. coli* in the presence of Alexa 488 labeled RNA scaffold.

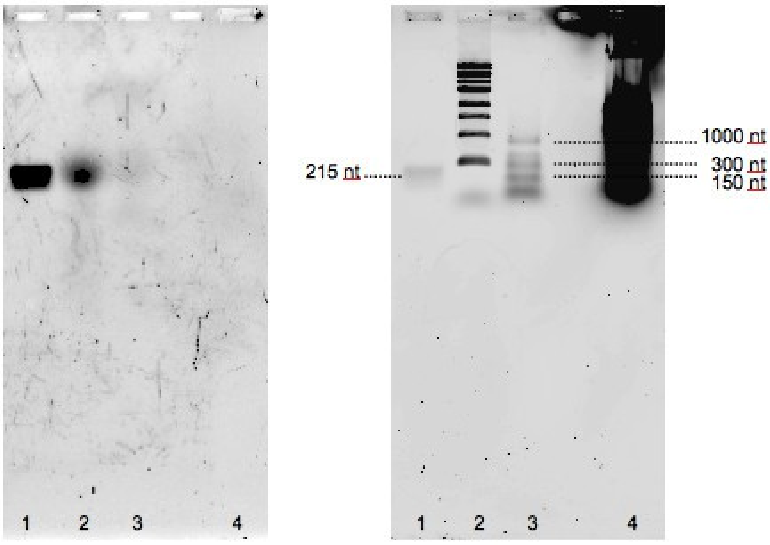


**Figure S17.** Agarose gel electrophoresis of Alexa 488 labeled noDBS1 sequence electroporated in *E. coli*. Cells were recovered for 25 minutes in SOC medium at 37 °C. After embedding in agarose plugs, *E. coli* cells were lysed, run on a standard agarose gel and imaged using Typhoon laser scanner before (excitation 488 nm; emission 532 nm, left) and after (right) SYBR® Gold staining. Lanes. 1: Alexa 488 scaffold noDBS1; 2: 1 Kb Quick Load DNA ladder; 3: ssRNA ladder; 4: electroporated *E. coli* in the presence of Alexa 488 labeled noDBS1 RNA scaffold. Note: lane 2 on left image (1Kb Quick Load DNA ladder) showed a spot due to the loading dye.

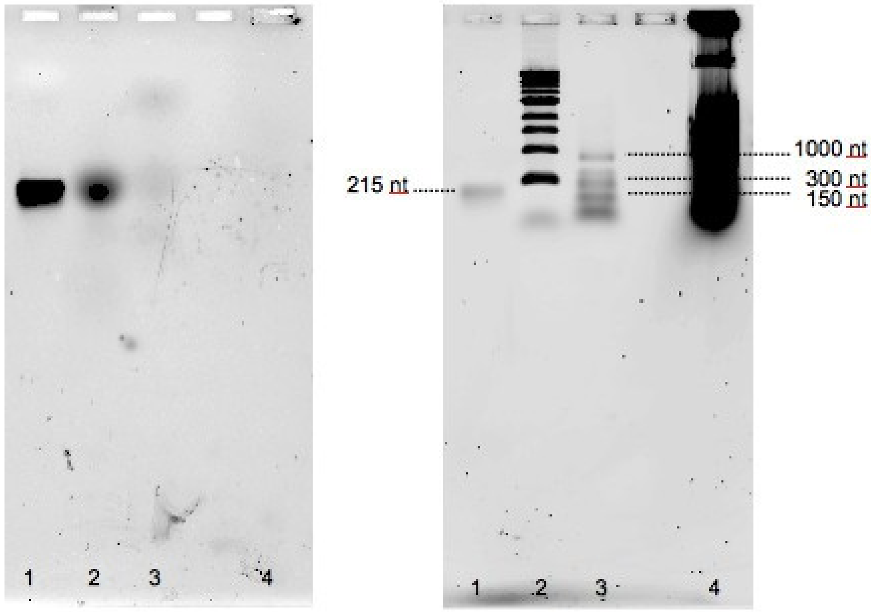


**Figure S18.** Agarose gel electrophoresis of Alexa 488 labeled noDBS1 sequence electroporated in *E. coli*. Cells were recovered for 25 minutes in SOC medium at 37 °C. After embedding in agarose plugs, *E. coli* cells were lysed, run on a standard agarose gel and imaged using Typhoon laser scanner before (excitation 488 nm; emission 532 nm, left) and after (right) SYBR® Gold staining. Lanes. 1: Alexa 488 scaffold noDBS2; 2: 1 Kb Quick Load DNA ladder; 3: ssRNA ladder; 4: electroporated *E. coli* in the presence of Alexa 488 labeled noDBS2 RNA scaffold. Note: lane 2 on left image (1Kb Quick Load DNA ladder) showed a spot due to the loading dye.
